# Traffic Crash Severity Prediction—A Synergy by Hybrid Principal Component Analysis and Machine Learning Models

**DOI:** 10.3390/ijerph17207598

**Published:** 2020-10-19

**Authors:** Khaled Assi

**Affiliations:** Civil & Environmental Engineering Department, King Fahd University of Petroleum & Minerals, Dhahran 31261, Saudi Arabia; khaledassi@kfupm.edu.sa

**Keywords:** traffic crash severity, vehicle crashes, emergency management, principal component analysis (PCA), neural networks (NN), support vector machine (SVM)

## Abstract

The accurate prediction of road traffic crash (RTC) severity contributes to generating crucial information, which can be used to adopt appropriate measures to reduce the aftermath of crashes. This study aims to develop a hybrid system using principal component analysis (PCA) with multilayer perceptron neural networks (MLP-NN) and support vector machines (SVM) in predicting RTC severity. PCA shows that the first nine components have an eigenvalue greater than one. The cumulative variance percentage explained by these principal components was found to be 67%. The prediction accuracies of the models developed using the original attributes were compared with those of the models developed using principal components. It was found that the testing accuracies of MLP-NN and SVM increased from 64.50% and 62.70% to 82.70% and 80.70%, respectively, after using principal components. The proposed models would be beneficial to trauma centers in predicting crash severity with high accuracy so that they would be able to prepare for appropriate and prompt medical treatment.

## 1. Introduction

RTCs represent a critical public health problem worldwide. Peden et al. [1] stated that RTCs are a major cause of serious injuries and fatalities in many countries. According to the WHO global status report on traffic safety, 1.35 million people die yearly as a result of traffic crashes, and traffic crash injuries are the main reason for deaths among young people. Moreover, it is reported that road crashes are the eighth major cause of death [2]. Andersson and Menckel [3] concluded that, to identify the primary reasons for the crashes and ways of effective prevention, it is important to understand the nature of the traffic safety matrix. RTC severity is of considerable interest to policymakers and safety specialists because of its significant economic and social impacts on society. The accurate prediction of traffic crash severity contributes to generating crucial information, which can be used to adopt appropriate measures for reducing the aftermath of crashes. Moreover, accurate severity prediction of traffic crashes can help hospitals to provide medical care quickly if a crash occurs. Many models have been proposed to predict RTC severity using environmental, crash, vehicle, driver, and roadway attributes. Among these models, multinomial logit models (MNL), probit logit models (PL), ordered probit models (OP), Bayesian networks (BN), neural network (NN) models, and SVM are widely used [4].

The heterogeneity and multidimensionality of RTC datasets made it difficult for researchers to analyze and identify the hidden relationships between the crash factors. PCA is a multivariate analysis technique that analyzes multidimensional datasets to extract the important information from such datasets and reduce its dimensionality without losing a lot of information while retaining variation as much as possible [5]. This study aims to develop a hybrid system combining PCA with MLP-NN and SVM in predicting RTC severity. The novelty of the paper is to investigate the performance enhancement due to the adoption of PCA for NN and SVM. This study is not focusing on feature reduction rather on the performance of the models. This study is particularly important for the appropriate management of crashes as this approach-based tool can easily help to determine the right kind of severity and take required health care measures and ensure the right apportionment of resources, especially during resource scarcity. The proposed models would be beneficial to trauma centers in predicting crash severity with acceptable accuracy so that they would be able to prepare for appropriate and prompt medical treatment. The results of this study revealed that using principal components caused a significant enhancement in the severity prediction accuracy and F1 score of MLP-NN and SVM models for both severity level (i.e., serious/fatal and slight).

The remaining sections of the paper are organized as follows: a literature review related to traffic crash severity prediction is given in Section 2. The methodology of this study is explained in Section 3. Section 4 presents data processing in which the MLP-NN and SVM models were developed. Results and discussions are explained in Section 5. Lastly, Section 6 provides the conclusions of this paper.

## 2. Literature Review

Studies about RTC severity prediction have been conducted widely worldwide. The methods followed in these studies varied from the traditional statistical methods to sophisticated machine learning (ML) methods. Shanker et al. [6] used MNL in their study to predict traffic crash severity in Washington state, USA. It was found that using MNL is a promising approach to predict traffic crash severity. Garrido et al. [7] developed an OP model to predict crash severity in Coimbra, Portugal. The analysis results revealed that the accident type has a significant impact on crash severity levels. In another study [8], the classification and regression trees (CART) model was used for crash severity prediction in Iran. The effect of reducing the three-class severity levels to two-class binary labels on prediction performance was investigated. It was found that using binary class severity labels caused a significant improvement in the prediction performance of CART. Oña et al. [9] used crash severity levels to classify traffic crashes in Granda, Spain. The authors used 18 crash attributes in developing a BN model. Several measures were used to evaluate the developed model such as accuracy, sensitivity, and specificity. It was found that the prediction accuracy of the developed models ranged from 57% to 59%, which represents low accuracies. Zong et al. [10] investigated the use of BN and regression models in RTC severity prediction in Jilin, China. A comparison between the goodness of fit of both proposed models was conducted. It was found that BN outperformed the regression models in crash severity prediction.

As an approach to enhance the prediction performance of traffic crash severity, many researchers tried to employ ML models. Abdelwahab and Abdel-Aty [11] used two NN models namely, MLP and fuzzy adaptive resonance theory (ART), in RTC severity prediction in the Central Florida area, USA. The developed NN models’ performance was compared with that of the OL model. It was found that MLP was superior when compared with ART and OL. Alkheder et al. [12] developed a NN model for the severity prediction of RTC in Abu Dhabi, UAE. A k-means clustering algorithm was used to cluster the crash dataset into three clusters. The analysis results revealed that the prediction accuracy was improved significantly after clustering. Moreover, when compared with the prediction performance of OP, the developed NN model was found to be superior with an accuracy of 74.6%. Zheng et al. [13] investigated the use of convolutional neural networks (CNN) for RTC severity prediction. A comparison was conducted between the proposed CNN model and nine common statistical and ML models such as k-nearest neighbor (KNN), decision trees (DT), SVM, and NN based on crash severity prediction performance. The comparison results revealed that CNN outperformed the other models in traffic crash severity prediction with an average F1score of 84%. Hashmienejad and Hashmienejad [14] proposed a multi-objective genetic algorithm to predict crash severity in Tehran, Iran. A comparison was conducted between the proposed method and other common models used in crash severity prediction such as NN, SVM, and conventional DT. The evaluation results revealed that the proposed method was superior with a prediction accuracy of 88.2%. Zeng and Haung [15] investigated the effect of NN model optimization on crash severity prediction in Florida, USA. A function approximation algorithm and a convex combination algorithm were proposed to optimize the network structure and its prediction capability. The proposed NN model was compared with another NN model trained by the backpropagation algorithm and the OL model. It was found that the developed model outperformed the other models in severity prediction and training time. Arhin and Gatiba [16] developed a NN model to predict RTC severity at un-signalized intersections in Washington DC. A systematic approach was followed to find the optimal topology of the NN model. The optimal architecture was selected based on prediction accuracy. It was found that the architecture resulted in the highest prediction accuracy (85.62%) had three hidden layers with 5, 10, and 5 neurons in the first, second, and third layers respectively. Although the attained accuracy was high, it should be mentioned that the authors used two severity levels which are injury crash and non-injury crash which means that they combined fatal/serious crashes and slight crashes in one class.

Many comparative studies were conducted worldwide to compare the performance of different models when used for traffic crash severity. Abdel-Aty and Abdelwahab [17] conducted a study to predict injury severity levels for traffic crashes in the Central Florida area. The study aimed to make a comparative analysis between three models namely, MLP-NN, ART, and the traditional OP model. In this study, the authors started using all input variables in developing the NN models. Then, a systematic procedure was followed to reduce the number of input variables. The model that resulted in the highest prediction accuracy was selected as the best model. The results revealed that NN models were superior when compared with the other models. Iranitalab and Khattak [18] made a comparison between MNL, random forest (RF), SVM, and nearest neighbor classification (NNC) based on crash severity prediction performance in Nebraska, USA. Moreover, the effect of k-means clustering and latent class clustering on the prediction power of the proposed models was investigated. The accuracy results revealed that NNC outperformed the other models in predicting most of the severity levels. Moreover, it was found that the effect of clustering on the prediction performance of the models was inconsistent. Zhang et al. [19] made a comparison between various statistical and ML methods based on crash severity prediction performance in Florida, USA. Two statistical methods (OP and MNL) and four ML methods (KNN, DT, RF, and SVM) were developed and compared. Based on accuracy results, it was found that RF outperformed the other models in crash severity prediction with a testing accuracy of 53.90%. Wang and Kim [20] conducted a study to compare discrete choice and tree-based models based on RTC severity prediction. In their study, they developed two models namely MNL and RF. Prediction accuracy was used to compare the performance of both models in terms of RTC severity prediction. It was found that the RF model outperformed the MNL model. In their study, Cuenca et al. [21] made a comparison between different ML techniques, such as deep learning, naïve Bayes, and gradient boosted trees, based on their prediction performance of RTC severity. Prediction accuracy results revealed that the deep learning model outperformed the other models. Sohn and Shin [22] conducted a study in Korea about traffic crash severity prediction. NN, DT, and logistic regression models (LR) were developed. The effect of variable reduction was investigated in this study. The three developed models were compared based on their prediction accuracy. The results revealed the prediction accuracies were not significantly different. Moreover, it was found that variable reduction was effective in enhancing the prediction accuracy.

Li et al. [23] conducted a study to predict traffic crash severity using SVM in Florida, USA. The prediction accuracy of SVM was compared with that of the OP model. It was found that the accuracy of SVM (48.8%) was higher than that of the OP model (44.0%). Although the accuracy was found to be very low, the authors did not apply any dimension reduction technique such as principal component analysis (PCA) on the crash dataset to overcome the problems of correlation between the input variables. Fiorentini and Losa [24] investigated the effect of applying balancing techniques on crash data on the performance of multiple ML models such as random tree, KNN, LR, and RF. It was found that introducing balancing technique enhanced the prediction power of the developed models. However, the attained accuracies were very low (~ <60%). In their study, Zhang et al. [25] developed an improved NN model to predict RTC severity by utilizing particle swarm optimization (PSO) method to optimize the structure of NN. The results revealed that the increase in prediction accuracy after introducing PSO was not significant. Hasheminejad et al. [26] proposed a new rule-based genetic algorithm model to predict RTC severity on rural roads. A comparison was conducted between the proposed model and other models such as NN, SVM, and k-NN based on prediction accuracy. It was found that the proposed model outperformed the other models with an accuracy of 87%. Assi et al. [27] conducted a study to investigate the effect of clustering on the performance of NN and SVM in predicting RTC severity. The models were compared based on accuracy and F1 score. It was found that the clustering algorithm increased the prediction accuracy of NN and SVM models from 70% and 73% to 71% and 74%, respectively. Despite this increase in accuracy, still, the attained accuracy was low which is maybe due to the correlation between the input variables. Based on the results of the previous study, the author of this study tried to enhance the prediction accuracy of RTC severity by investigating the effect of dimension reduction (by using PCA) of a crash dataset on the performance of NN and SVM models in RTC severity prediction. PCA was used by scholars to find out the important features affecting crash severity [28,29,30].

It can be observed from the literature survey that many attempts were conducted to predict RTC severity using different statistical and ML algorithms. The prediction accuracy of the developed methods was generally low, which is most probably due to the high correlation between crash attributes. Moreover, PCA was used mainly in previous studies to find the important features that affect crash severity. Most of the previous works focused on model performance without much considerations for the availability of the data. Moreover, previous studies lack the engineering practicality. Hence, this study aimed to develop a model which would be beneficial in saving lives by predicting crash severity with acceptable accuracy based on the crash features, which can be easily obtained from the crash location. Based on this information, the trauma centers would be able to prepare for appropriate and prompt medical treatment. Hence, this study aims to apply PCA, which is seldom used in such studies, on the crash dataset and to investigate its effect on crash severity prediction performance of two commonly used ML models for crash severity prediction (as found in the literature), namely MLP-NN and SVM models.

## 3. Methodology

### 3.1. Dataset Discerption

The dataset for traffic crashes that occurred in Victoria, Australia for five years (2014 to 2019) was used in this study with a total number of crashes of 74,909. The data was collected from the department of transport open data website. Only two-vehicle and single-vehicle crashes were considered in this study. After preprocessing the dataset (i.e., deleting incomplete cells, unknown values, and repeated records) and excluding the crashes in which more than two vehicles were involved, the number was decreased to 37,774 crashes. Each crash in the dataset is represented by 16 attributes, including crash, road, driver, vehicle, and environmental characteristics, as shown in Table 1. The crash severity in the original dataset comprised three levels: fatal, serious injury, and slight injury with frequencies of 776 (2.05%), 14,237 (37.69%), and 22,761 (60.26%), respectively. As a result of the limited number of fatal crashes, it was decided to aggregate fatal and serious injury severity levels as done in some previous studies [18,22]. Hence, the new severity levels are serious/fatal injury and slight injury.

### 3.2. Multilayer Perceptron Neural Networks

**Figure 1:**
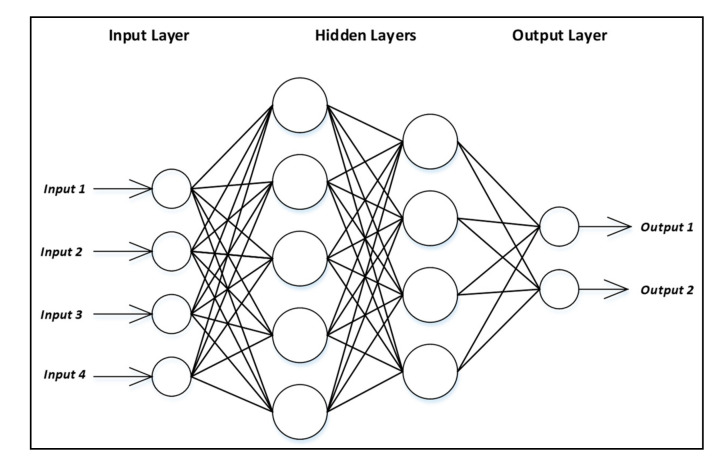
Simplified structure of MLP-NN.

MLP-NNs are a set of algorithms in which human intelligence is incorporated into computing machines that can efficiently capture and represent extremely complex nonlinear relationships with better generalization performance [31,32]. The capabilities of fast operation, parallel computation, and ease of implementation make the MLP-NN widely used by researchers. MLP-NNs are constructed mainly from an input (raw data receiver), output (outcome displayer), and hidden neuron layers where the nodes of a layer and next layer (if any) are connected entirely as shown in Figure 1. Each node applies non-linear activation functions to process the incoming inputs and then transfers the outputs to the subsequent layer’s nodes.

The number of neurons in the input layer equals the number of inputs while in the output layers equals to the number of outputs. To determine the number of neurons in the hidden layer, trial and error procedure or any other optimization method [33] can be followed. The output of one layer is the input of the next layer. Every node in a layer is connected entirely with all nodes of the previous layer. The weights on these connections encode the knowledge of a network. These weights can be estimated using optimization methods by minimizing the loss function.

### 3.3. Support Vector Machine

**Figure 2:**
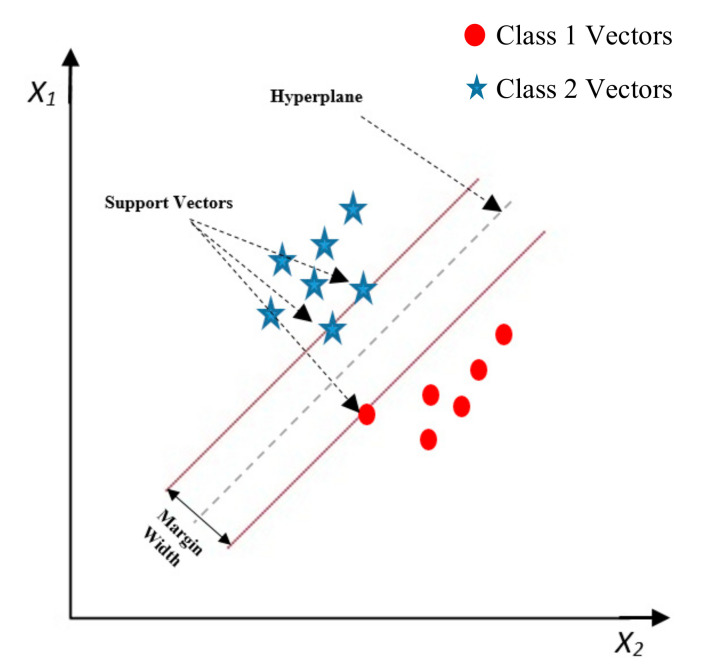
Data separation by hyperplanes.

SVM is a supervised machine learning algorithm developed by Vapnik et al. [34,35], which can be used for classification or regression problems. As a result of its high generalization performance, SVMs are widely used for high dimensional data analysis [36,37,38]. In the regression type function, SVM optimally separates the output classes by constructing hyperplanes [39], which represent decision boundaries that classify data points into their respective classes. Figure 2 shows a hyperplane separating two classes of support vectors. As shown in Figure 2, the closest points to the hyperplane represent the support vectors.

All crash input variables in SVM are represented by vectors (*x_i_ ∊ R^n^*) for [*i* = 1,2,3,…*N*] and RTC severity which represents training output is represented as (*y_i_ ∊ R^n^*). The following formula (Equation (1)) represents the hyperplane which separates the outcomes as a set of point *X*;

*W·X* − *b* = 0 (1)

where vector *W* denotes the normal vector, which is perpendicular to the hyperplane. SVM should be optimized in a binary classification problem for a given set of input and output variable pairs (*x_i_*, *y_i_*) as shown in Equations (2) and (3).

(2) $$min_{w,b,\xi }\;\frac{1}{2}w^{T}w+C\overset{N}{\underset{i=1}{\sum }}\xi_{i}$$

(3) $$\mathrm{Subject}\;\mathrm{to}\;y_{i}(w^{T}\phi \left(x_{i}\right)+b\geq 1-\xi_{i},\;\xi_{i}\geq 0.$$

where *ξ* are slack variables that measure misclassification errors and *C* is the penalty factor to errors that enhance the capacity control of the classifier.

It should be mentioned that by increasing the margin width, the classification of the data by hyperplane would be more accurate. Different kernels can be used to create the separation surface, such as radial basis, polynomial, and sigmoidal functions [40].

### 3.4. Principal Component Analysis

Dealing with large multidimensional datasets such as crash datasets is sometimes difficult and time-consuming. To analyze such datasets, some techniques such as PCA are required to reduce their dimensionality. The PCA is one of the most widely used multivariate statistical techniques and was originally developed by Hotelling [41]. Orthogonal transformation is used in PCA to convert a set of correlated variables into a set of score values of linearly uncorrelated variables called principal components without significant loss of information [42]. A significant amount of research has been carried out with respect to the application of PCA. However, PCA use in traffic safety is relatively new. Suppose the random vector *X* = [*X_1_*, *X_2_*, …., *X_n_*] has the covariance matrix V with eigenvalues *β_1_* ≥ *β_1_* ≥ ……, *β_n_* ≥ 0 and normalized eigenvectors *l_1_*,*l_2_*,…., *l_n_*. Consider the linear combination:

(4) $$X_{PC_{i}}=l_{i}^{t}X=l_{1i}X_{1}+l_{2i}X_{2}+\cdots \;l_{ni}X_{n}$$

(5) $$Var\;\left(X_{PC_{i}}\right)=l_{i}^{t}Vl_{i},\;i=1,2,\ldots \ldots ,n,$$

(6) $$Cov\;\left(X_{PC_{i}},X_{PC_{k}}\right)=l_{i}^{t}Vl_{k},\;k=1,2,\ldots ,n$$

where *t* represents the transpose operator. The uncorrelated linear combinations $X_{PC_{1}},X_{PC_{2}},\ldots ,X_{PC_{n}}$ represent the principal components ranked in descending order based on their variances [43].

## 4. Data Processing

In this study, the Python environment was used to develop and testify machine learning models and to conduct PCA. Before feeding the data into ML models, all categorical variables were converted to binary values, and then, all variables in the dataset were normalized and scaled into a range of [0, 1] to improve the performance. Two machine learning models were considered in this study, MLP-NN and SVM. Each model was developed two times. In the beginning, the models were developed using the original crash attributes shown in Table 1 as input variables. Then, the same models were developed using the principal components found after applying PCA. In developing the models, it should be mentioned that 70% of the dataset was used for training while the remaining 30% was used for testing [44].

### 4.1. Development of the Multilayer Perceptron Neural Networks Model Using Original Crash Attributes

The number of the input nodes used was equal to the number of crash attributes. The optimal topology of the model was determined by following a systematic procedure starting with one hidden layer with a small number of neurons. The number of hidden layers and their neurons was then increased systematically until a high accuracy was achieved without overfitting. Overfitting can be observed by comparing the classification accuracy of the testing dataset with that of the training dataset. If the model performs much better on the training dataset than on the testing dataset, it means that the model is likely overfitting. The best training function was found to be Bayesian regularization backpropagation as it resulted in the maximum prediction accuracy compared with the other training algorithms, such as variable learning rate backpropagation, resilient backpropagation, Levenberg–Marquardt, BFGS quasi-Newton, and scaled conjugate gradient.

The optimal topology of the MLP-NN model, which resulted in the maximum prediction accuracy was found to be two hidden layers with ten neurons in the first hidden layer (with tangent sigmoid activation function) and two neurons in the second layer (with Softmax activation function).

### 4.2. Development of the Support Vector Machine Model Using Original Crash Attributes

In developing any SVM model, it is important to find the optimal values of the penalty parameter (C) and kernel scale gamma (γ). The C parameter represents the trade-off between training error and the flatness of the solution while the γ parameter which explains the effect of a single training example. In this study, the procedure suggested by Chang and Lin [45] was followed in determining the values of C and γ parameters. According to this procedure, small and large values of C should be tried. Then, the value of C which results in better performance should be selected. For the selected C, different values of γ should be tried to find the best value.

It was found that the best values of C and γ for the developed SVM model were 0.6 and 5 respectively. The Gaussian radial basis kernel function resulted in the best classification accuracy compared to other kernels such as the polynomial kernel, the Laplace RBF kernel, the hyperbolic tangent kernel, and the sigmoid kernel.

### 4.3. Principal Component Analysis

Before proceeding with the PCA, two tests were conducted on the available dataset to check its suitability for multivariate analysis. These tests are the Kaiser–Meyer–Olkin (KMO) test and Bartlett’s test of sphericity [42]. The KMO test (a measure of sampling adequacy) was used to detect the multicollinearity in the available dataset so the appropriateness of carrying out a PCA could be detected. When the value of KMO approaches the maximum value (which is 1), it indicates that a high correlation exists among the variables of the dataset, which justifies using PCA.

**Figure 3:**
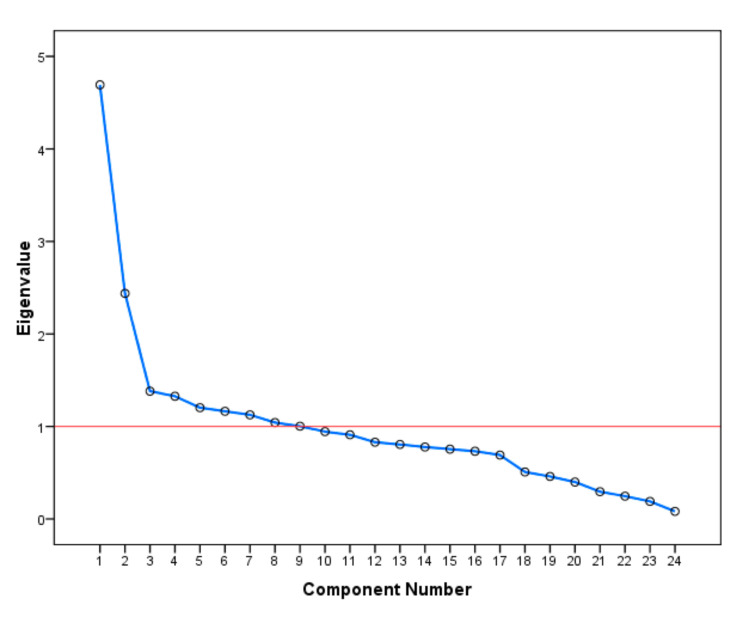
Eigenvalues for all components considered (scree plot).

Barlett’s test of sphericity assesses the hypothesis that the dataset variables are unrelated and are, therefore, unsuitable for PCA [42] as PCA is suitable when only there is a high correlation between the dataset variables. In this study, Python was used to conduct these two tests on the available crash dataset. It was found that the KMO value was 0.79 and the *p*-value for Bartlett’s test of sphericity was 0, which indicates that PCA is suitable for our crash dataset. Hence, PCA has conducted on the normalized crash dataset considered in this study using Python. The main target of PCA was to find the principal components (PCs) that can represent the original dataset with a minimum loss of information. The maximum number of components is equal to the number of original attributes. Several methods can be followed to determine the number of PCs. In this study, the Kaisar-Guthman rule [46] was opted to select the number of PCs. According to this rule, the optimal number of PCs is equal to the number of components having an eigenvalue (the variance explained by each component) greater than one. The scree plot shown in Figure 3 shows the eigenvalues for all components considered in the PCA.

**Figure 4:**
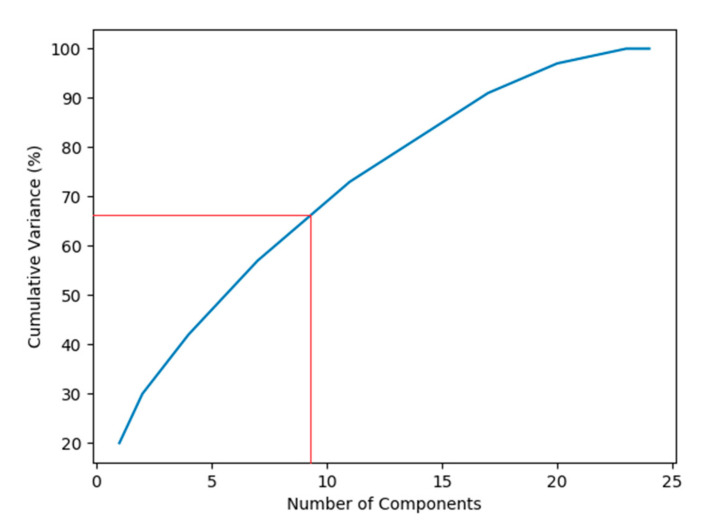
Cumulative variance plot.

It can be observed from Figure 3 that the first nine principal components have an eigenvalue greater than one. Therefore, these nine components represent the major principal components of the crash dataset used in this study. The cumulative variance percentage explained by these principal components (the first nine components) was found to be 67%, as shown in Figure 4.

After finding the major principal components, each crash in the dataset can be represented by nine components instead of the original 24 attributes shown in Table 1. The highly correlated original features for the nine principal components are crash type, road surface condition, traffic control type, driver’s gender, vehicle type, road surface type, roadway speed, road geometry, and driver’s age. Most of these variables were found to be significant in predicting RTC severity in previous studies, as shown in Table 2.

### 4.4. Development of the Multilayer Perceptron Neural Networks and Support Vector Machine Models Using Principal Components

In this section, the principal components dataset found in the previous section was used instead of the original crash dataset in developing new MLP-NN and SVM models to investigate the effect of using principal components on the severity prediction performance of these models. The same procedures mentioned in Section 4.1 and Section 4.2 were followed in developing the models. The optimal topology of the MLP-NN model, which resulted in the maximum prediction accuracy was found to be two hidden layers with six neurons in the first hidden layer (with tangent sigmoid activation function) and three neurons in the second layer (with Softmax activation function). The best training function was found to be Bayesian regularization backpropagation.

Regarding the SVM model, the best values for the C and γ parameters after using the principal components were found to be 1 and 15, respectively, and the best kernel function was found to be the Gaussian radial basis kernel function.

## 5. Results and Discussion

**Figure 5:**
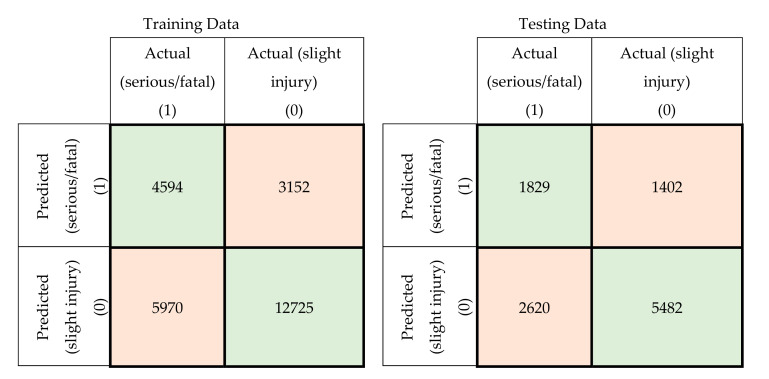
Confusion matrices for the MLP-NN model using original crash attributes (training and testing data).

**Figure 6:**
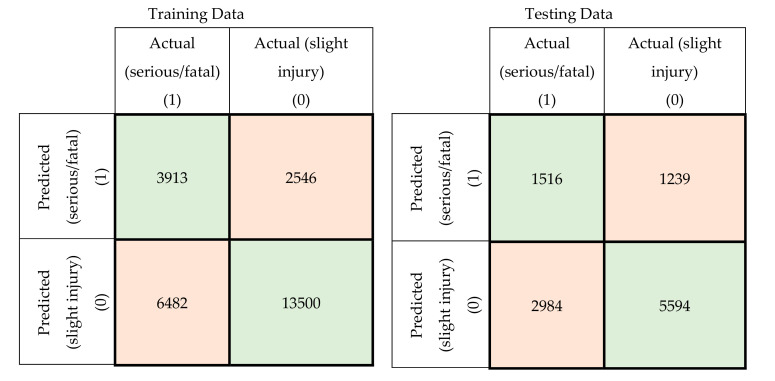
Confusion matrices for the SVM model using original crash attributes (training and testing data).

**Figure 7:**
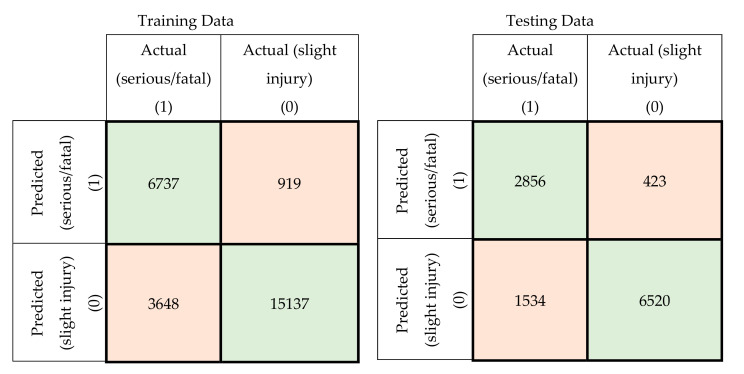
Confusion matrices for the MLP-NN model using principal components (training and testing data).

**Figure 8:**
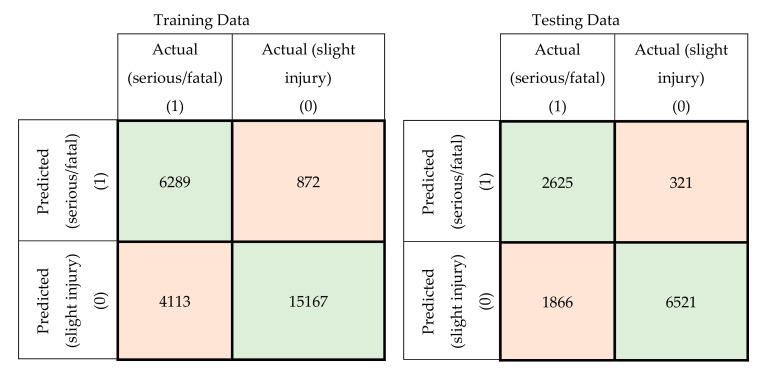
Confusion matrices for the SVM model using principal components (training and testing data).

In this section, a comparison was conducted between the prediction accuracy and F1 scores of the models developed using the original attributes with those of the models developed using principal components. The confusion matrices for the training and testing datasets for the developed MLP-NN and SVM models using original attributes are shown in Figure 5 and Figure 6, respectively. The confusion matrices for the same models developed using principal components are shown in Figure 7 and Figure 8.

A comparison was conducted between the developed models based on classification accuracy and F1 score. Classification accuracy is the proportion of the observation predicted correctly to the total number of observations as shown in Equation (7) while the F1 score is the harmonic mean of precision and sensitivity, which can be found using Equation (8) [50].

(7) $$Accuracy=\frac{Number\;of\;observations\;predicted\;correctly}{total\;number\;of\;observations}$$

(8) $$F1\;score=\frac{2*precision*sensitivity}{\left(precision+sensitivity\right)}$$

where sensitivity is the ratio of the number of crashes correctly predicted as severe/non-severe to the total number of actual severe/non-severe crashes, which can be expressed using Equations (9) and (10). Precision is the ratio of the number of crashes correctly predicted as severe/non-severe to the total number of predicted severe/non-severe crashes, which can be expressed using Equations (11) and (12).

(9) $$Sensitivity\;\left(serious/fatal\right)=\frac{\#of\;crashes\;correctly\;predicted\;as\;serious/fatal}{Total\;actual\;serious/fatal\;crashes}$$

(10) $$Sensitivity\;\left(slight\right)\;=\;\frac{\#of\;crashes\;correctly\;predicted\;as\;non-severe}{Total\;actual\;nono-severe\;crashes}$$

(11) $$Precision\;\left(serious/fatal\right)=\;\frac{\#of\;crashes\;correctly\;predicted\;as\;serious/fatal}{Total\;predicted\;serious/fatal\;crashes}$$

(12) $$Precision\;\left(slight\right)=\;\frac{\#of\;crashes\;correctly\;predicted\;as\;nono-severe}{Total\;predicted\;non-severe\;crashes}$$

The training and testing classification accuracies, sensitivity ratios, precision ratios, and F1 scores for all developed models for serious/fatal and slight crashes are summarized in Table 3 and Table 4, respectively.

**Figure 9:**
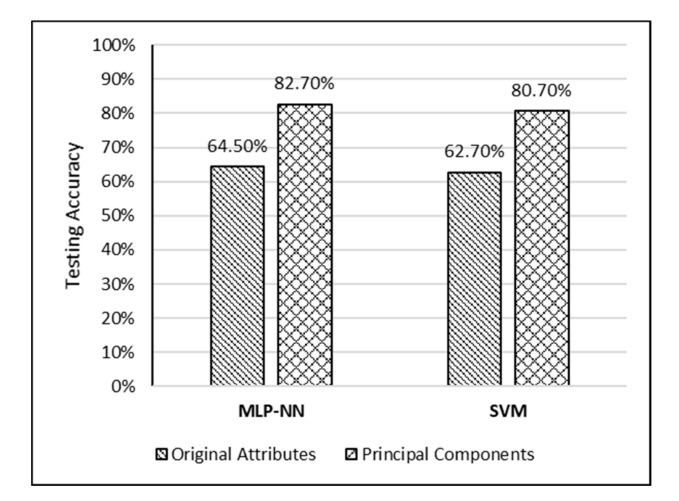
Testing classification accuracies for the developed models.

**Figure 10:**
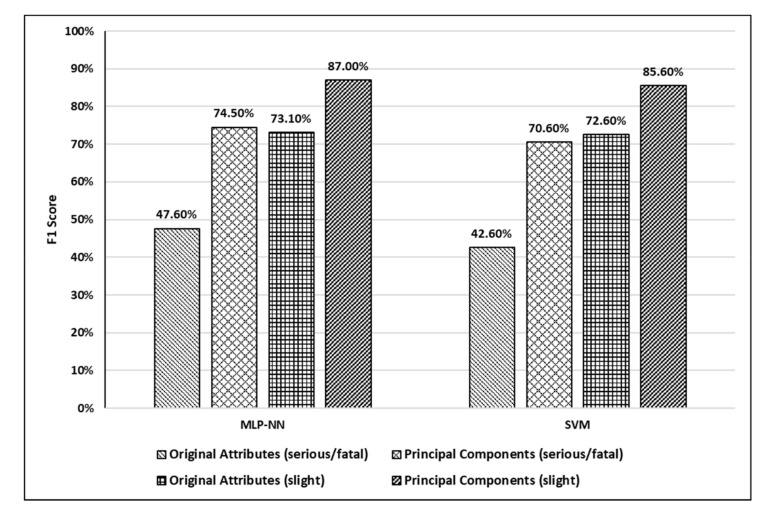
F1 scores for the developed models (serious/fatal injury and slight injury).

For comparison purposes, testing accuracies and F1 scores for the developed models are shown in Figure 9 and Figure 10, respectively.

Based on the accuracy results shown in Figure 9, it can be observed that MLP-NN performed slightly better than SVM when original attributes were used. Moreover, it can be observed that using principle components enhanced the prediction accuracy of both models significantly. Using accuracy only for comparison in such studies is misleading due to highly imbalanced crash severity distributions. Hence, the F1 score was used as another comparison measure in this study as shown in Figure 10. It can be observed from Figure 10 that both models (MLP-NN and SVM) performed very well in predicting the severity of slight injury crashes compared to serious/fatal injury crashes in both scenarios (using original attributes and using principal components). Moreover, using principles components resulted in a significant jump in the F1 scores of both models. The increase in F1 score values after using principal components was more significant in the case of serious/fatal crashes.

The prediction accuracy of the proposed hybrid system was found to be higher than the prediction accuracies of previous studies in which the authors applied neural networks or support vector machines, which are shown in Table 5.

## 6. Conclusions

Traffic crash severity is of considerable interest to policymakers and safety specialists because of its significant economic and social impacts on society. The accurate prediction of traffic crash severity contributes to generating crucial information, which can be used to adopt appropriate measures for reducing the aftermath of crashes. Moreover, accurate severity prediction for traffic crashes can help hospitals to provide medical care quickly if a crash occurs. This study aimed to investigate the effect of using principal component analysis on the performance of MLP-NN and SVM in predicting traffic crash severity. The dataset comprised traffic crashes that occurred in Victoria, Australia for five years (2014 to 2019). The total number of crashes reported during this period was 74,909. Only two-vehicle and single-vehicle crashes were considered in this study. After preprocessing the dataset (i.e., deleting incomplete cells, unknown values, and repeated records) and excluding the crashes in which more than two vehicles were involved, the number was decreased to 37,774 crashes.

Python was used to develop and test the machine learning models considered in this study and to conduct PCA. Based on the PCA results, it was found that the first nine components have eigenvalues greater than one. Therefore, these nine components represent the major principal components of the crash dataset used in this study. The percentage of cumulative variance explained by the principal components (the first nine components) was found to be 67%. Two machine learning models were considered in this study: MLP-NN and SVM. Each model was developed twice. The original crash attributes shown in Table 1 were used in the first trial as input variables while the principal components found after applying PCA were used in the second trial. The prediction accuracies and F1 scores of the models developed using the original attributes were compared with those of the models developed using principal components. It was found that the testing accuracies and F1 scores of MLP-NN and SVM increased significantly after using principal components, which represents a significant enhancement. Moreover, the prediction accuracy of the proposed hybrid system was found to be higher than the prediction accuracies of previous studies about traffic crash severity prediction in which the authors applied neural networks or support vector machines. Furthermore, it should be mentioned that PCA was able to simplify the problem with a reduction in dimensionality which resulted in significant improvement in traffic crash severity prediction. The proposed models would be beneficial in saving lives by predicting crash severity with acceptable accuracy based on the crash features, which can be easily obtained from the crash location. Based on this information, the trauma centers would be able to prepare for appropriate and prompt medical treatment

### Limitations and Future Work

A systematic approach was followed in this study in determining the architecture of MLP-NN and C and gamma values for SVM. The authors could have used advanced optimization functions, such as genetic algorithm and practical swarm optimization, to find the optimal architecture of MLP-NN and optimal C and values of SVM. Moreover, advanced data balancing approaches could be used in this study to balance the dataset. As future work, it is recommended to investigate the impact of PCA and other data reduction techniques on other ML models, such as deep learning, random forest, and decision trees, when used for traffic RTC severity prediction.

## Figures and Tables

**Table 1:** Crash, driver, vehicle, and environmental characteristics.

Category	Variable	Description
Crash Characteristics	Crash type	1. Collision with a vehicle; 2. Struck pedestrian; 3. Struck animal; 4. Collision with a fixed object; 5. Collision with some other object; 6. The vehicle overturned; 7. Fall from or in moving vehicle; 8. Other crash.
	Day of the week	1. Monday; 2. Tuesday; 3. Wednesday; 4. Thursday; 5. Friday; 6. Saturday; 7. Sunday
	Number of vehicles involved	Integer value (with a maximum of two vehicles)
	Number of persons involved	Integer value
Roadway Characteristics	Roadway median separation	0. Undivided, 1. Divided
	Roadway geometry	1. Cross intersection; 2. T intersection; 3. Y intersection; 4. Multiple intersections; 5. Not at an intersection; 6. Dead end; 7. Road closure
	Roadway speed	Integer value
	Roadway surface condition	1. Dry; 2. Wet; 3. Muddy; 4. Snowy; 5. Icy
	Roadway surface type	1. Paved; 2. Unpaved; 3. Gravel
	Traffic control	0. No control; 1. Stop-go lights; 2. Pedestrian lights; 3. Pedestrian crossing; 4. Roundabout; 5. Stop sign; 6. Give Way sign; 7. other
Environmental Characteristics	Weather condition	1. Clear; 2. Raining; 3. Snowing; 4. Fog; 5. Smoke; 6. Dust; 7. Strong wind
	Light condition	1. Day; 2. Dusk/Dawn; 3. Dark: streetlight on; 4. Dark: streetlight off; 5. Dark/no streetlights; 6. Dark/street lights unknown
Driver Characteristics *	Driver’s gender	0. Female; 1. Male
	Driver’s age	Integer value
Vehicle Characteristics *	Vehicle’s age	Integer value
	Vehicle type	1. Car; 2. A station wagon; 3. Utility vehicle; 4. Panel van; 5. Bus; 6. Motorcycle; 7. Moped, 8. Bicycle; 9. Quad bike

* For all drivers and vehicles involved in the crash.

**Table 2:** A highly correlated original feature for each principal component.

Principle Component No.	The Highly Correlated Original Feature
1	Crash Type [9]
2	Road Surface Condition
3	Traffic Control Type
4	Drivers’ Gender [47]
5	Vehicle Type [47,48]
6	Road Surface Type
7	Roadway Speed [47,48]
8	Road Geometry [47]
9	Driver’s Age [9,47,49]

**Table 3:** Performance measures of the developed models (serious/fatal injury crashes).

Model	Training Accuracy	Testing Accuracy	Sensitivity	Precision	F1 Score
MLP-NN with original attributes	65.5%	64.5%	41.1%	56.6%	47.6%
SVM with original attributes	65.9%	62.7%	34.7%	55.0%	42.6%
MLP-NN with principal components	82.7%	82.7%	65.1%	87.10%	74.5%
SVM with principal components	81.1%	80.7%	58.4%	89.1%	70.6%

**Table 4:** Performance measures of the developed models (slight injury crashes).

Model	Training Accuracy	Testing Accuracy	Sensitivity	Precision	F1 Score
MLP-NN with original attributes	65.5%	64.5%	79.6%	67.7%	73.1%
SVM with original attributes	65.9%	62.7%	81.8%	65.2%	72.6%
MLP-NN with principal components	82.7%	82.7%	93.9%	81.0%	87.0%
SVM with principal components	81.1%	80.7%	95.3%	77.8%	85.6%

**Table 5:** Findings of previous studies.

Study	Models	Prediction Accuracy
Abdelwahab and Abdel-Aty [11]	NN	60.4%
Alkheder et al. [12]	k-means clustering based NN	74.6%
Zeng and Haung [15]	NN trained by the convex combination algorithm	54.8%
Iranitalab and Khattak [18]	SVM	61.5%
Zhang et al. [19]	SVM	53.9%
Li et al. [23]	SVM	48.8%
Assi et al. [27]	Fuzzy c-means clustering-based SVM	74%
	Fuzzy c-means clustering-based NN	71%
